# Participatory Epidemiology of Ethnoveterinary Practices Fulani Pastoralists Used to Manage Contagious Bovine Pleuropneumonia and Other Cattle Ailments in Niger State, Nigeria

**DOI:** 10.1155/2015/460408

**Published:** 2015-03-23

**Authors:** N. B. Alhaji, O. O. Babalobi

**Affiliations:** ^1^Department of Veterinary Public Health and Preventive Medicine, Faculty of Veterinary Medicine, University of Ibadan, Ibadan, Nigeria; ^2^Public Health and Epidemiology Unit, Ministry of Livestock and Fisheries Development, Minna, Niger State, Nigeria

## Abstract

Ethnoveterinary practices are locally available and affordable to Fulani pastoralists in Niger State, Nigeria, to whom conventional veterinary services are often not readily available and are relatively expensive. This study was designed to identify and document medicinal plant and nonplant materials used by this group in the management of cattle diseases. Participatory rural appraisal tools of checklist, semistructured interview, probing, transect, and triangulations were used to assess Fulani pastoralists existing knowledge on traditional veterinary practices in nine pastoral communities spread across the state. Fifty medicinal materials and seven traditional preventive practices are in use against CBPP and other cattle disease conditions. Of these, 38 (76.0%) are medicinal plants and 12 (24.0%) are nonplant materials (edible earth materials and minerals). Family Fabaceae was most commonly mentioned while leaves were the most common parts used. Most of these materials are administered by drenching with few others mixed with feed. Proportions of plant parts used include leaves (47.4%), barks (31.6%), roots (10.6%), and 2.6% of each of rhizomes, fruits, seeds, and whole plants. Of recently used ingredients are kerosene and spent engine oil. Further research into the active ingredients of ethnoveterinary materials and dosages is necessary to guide their usage.

## 1. Introduction

For many years stock raising has been an important part of livelihood and culture in Sub-Saharan Africa [1–3]. The economic burden of livestock diseases and the declining provision of conventional veterinary services in this continent have undermined the efficiency of livestock production, especially by Fulani pastoralists [4]. Many people in developing countries still rely on medicinal plants and traditional healing practices for daily healthcare needs of their animals, in spite of the advancement in conventional medicine [5].

Conventional medical system, also called Western medicine, modern medicine, and biomedicine, used by most medical and veterinary doctors, focuses on disease as an enemy to be conquered. The conventional veterinary practitioner prescribes medications, uses the latest diagnostic tools, and follows peer-reviewed studies that could impact or change the way certain injuries or illnesses are treated. On the other hand holistic veterinary medicine includes such unconventional modalities as acupuncture, chiropractic, homeopathy, flower essences, raw diets, nutraceuticals (the use of concentrated doses of vitamins, minerals, and enzymes to treat disease), Chinese medicine, and herbs [6, 7].

There is abundant undocumented traditional knowledge of medicinal plants used to treat diseases in most cultures [8]. Different traditional healing practices worldwide are designed for either therapeutic or prophylactic use in human or animal diseases [9–11].

In Nigeria, pastoralists are known to treat animal diseases with herbs and other traditional medical practices before the advent of conventional medicine [12]. Traditional medical and veterinary practices remain relevant and vital in almost all cultures in Nigeria due to absence or inadequate provision of modern medical services especially in hard-to-reach rural areas [13]. Ethnoveterinary medical practice is widespread among pastoral herdsmen and village livestock keepers in northern Nigeria where most of the country's livestock are concentrated [14]. For most of these livestock owners, conventional veterinary inputs and services are not readily available and, where available, are relatively expensive. Therefore, they are left with traditional choices which are locally available and affordable, with the held belief that they are more efficacious [15].

In recognition of the fact that Fulani pastoralists possess considerable existing veterinary knowledge and traditional oral history of herbal and nonherbal remedies and their application in livestock disease management, veterinarians, recently, have intensified efforts towards harnessing this knowledge for authentication and preservation [16]. There is no record so far giving ethnoveterinary practices documentation in Niger State and there is likelihood that the practices are at the verge of extinction, especially among the Fulani pastoralists.

This survey was therefore aimed at assessing, in nonexperimental way, the ethnoveterinary practices used by Fulani pastoralists in Niger State to traditionally manage contagious bovine pleuropneumonia (CBPP) and other common cattle disease conditions in their herds. Also, herbal and nonherbal materials are to be identified, validated by consensus, and documented to add useful new remedies to the traditional veterinary pharmacopoeia.

## 2. Materials and Methods

### 2.1. Study Area

Niger State is located in the North-Central geopolitical zone, at the Northern Guinea Savannah ecological zone of Nigeria, between latitudes 8°20′N and 11°30′N and longitudes 3°30′E and 7°20′E. It is one of the 36 states of Nigeria, a gateway between Northern and Southwestern and South-Southern parts of the country, and provides transit routes for pastoral nomads on seasonal movements from the northern parts of Nigeria to the southern parts and back. The state covers a land area of about 76,363 square kilometers (29,484 square miles) or about 9% of Nigeria's total land area, making it the largest in terms of land mass in the country. The state has an estimated cattle population of about 2.4 million cattle, 1.7 million sheep, and 2.3 million goats in 2012 [17]. These cattle are in the custodies of nomadic and sedentary pastoralists.

**Figure 1:**
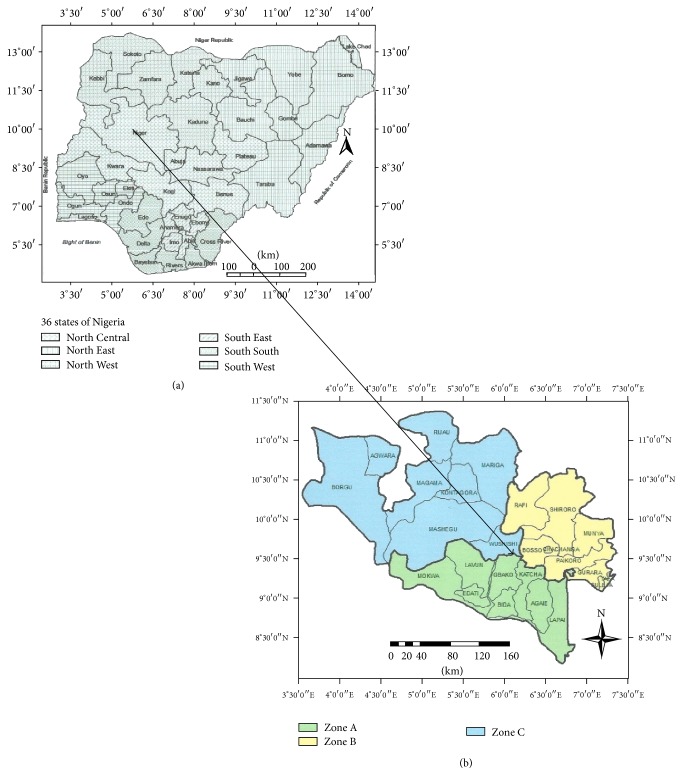
(a) Map of Nigeria showing location of Niger State. (b) Map of Niger State showing the three agroecological zones in the state.

The state shares a common international boundary with the Republic of Benin at its western border and has three agroecological zones, A (Bida zone), B (Minna zone), and C (Kontagora zone), which are based on different climatic conditions in the state [17] (Figure 1).

The research was conducted in the following pastoral communities: Lapai (GPS coordinate N09.0102° and E006.61729°); Eyagi (N09.13506° and E006.00618°); Lemu (N09.17155° and E006.01972°) in Agrozone A; Paiko (N09.43533° and E006.60745°); Kuta (N09.84643° and E006.71782°); Bosso (N09.66275° and E006.47691°) in Agrozone B; Wushishi (N09.69760° and E006.05682°); Bobi grazing reserve (N09.16715° and E005.91701°); Borgu (N09.91455° and E004.33400°) in Agrozone C.

### 2.2. Study Design

Participatory epidemiology (PE) exercise was conducted to collect qualitative data from the Fulani pastoralists in the nine communities using participatory rural appraisal (PRA) tools. The study focused on contagious bovine CBPP and other common disease conditions that frequently affect cattle in these communities and traditional remedies used to manage them. The survey was carried out between January and December, 2013.

### 2.3. Study Population

Fulani pastoralists in Lapai, Eyagi, Lemu, Paiko, Kuta, Bosso, Wushishi, Bobi grazing reserve, and Borgu pastoral communities were the population studied.

*Inclusion Criterion*. Only adult male Fulani pastoralists were considered because of their long time historical and sociocultural relationship with their cattle herds.

### 2.4. Sample Size

Three key informants were conveniently allocated to each of the nine pastoral communities for the purpose of the participatory exercises. Since nine pastoral communities were purposively selected, the number (sample size) of the key informants for the survey was, therefore, 27.

### 2.5. Sampling Procedure

Two-stage sampling method was used. In stage one, the state was divided into three sampling areas based on the existing three agroecological zones: A (Bida zone), B (Minna zone), and C (Kontagora zone) in the state. In stage two, three Fulani pastoral communities were conveniently selected in each agroecological zone by purposive sampling method. In addition to the key informants participation in the PE exercises for historical information about existing ethnoveterinary knowledge and practices on cattle diseases management, other pastoralists also participated in each community. However, the number of other participants in the exercises was not restricted since there was no size limit of attendance by others in each session.

### 2.6. Data Collection

The participatory rural appraisal (PRA) tools of key informants, checklist, semistructured interview, probing, transect, and triangulation [18–20] were used to discuss and collect information. Interviews and discussions were supplemented by “walk-in-the-woods” (transect) observations guided by key informants to identify and collect plant species, where necessary, for documentation. During participatory appraisal activities, informants were asked specific questions about the use of botanical and nonbotanical medicinal materials, methods of preparations, and applications.

The key informants' consensus factor on each plant or nonplant material used for a particular cattle disease condition gave indication of agreements on the usefulness of the material for such disease condition. An outline of the participants' initial ethnoveterinary remedies was drafted during each participatory session and further probed and discussed extensively in order to confirm the information provided. For every specimen identified the vernacular names were also recorded. The collected specimens were preserved and identified in the herbarium of Niger State Ministry of Agriculture and Natural Resources, Minna, Nigeria.

### 2.7. Data Analysis

Descriptive statistics of rates, charts, and tables were used. The collected ethnobotanical data and other ethnoveterinary information on CBPP and other cattle disease conditions were analyzed using the method of Friedman et al. [21] that expresses a plant's botanical efficacy by fidelity level. The fidelity level (key informants consensus) presents the most important plant species used for treating a particular cattle disease/condition as expressed by the key informants who are considered most knowledgeable elders possessing existing veterinary knowledge and traditional oral history on livestock in the pastoral communities. In this study, the fidelity level analytical approach was also used in evaluating the nonplants and prophylactic data generated during the participatory exercises. The fidelity level is mathematically expressed as FL = (*Ip*/*Iu*) × 100, where FL is the fidelity level of each plant or nonplant material, *Ip* is the number of key informants who mentioned that a plant or nonplant material has specific ethnoveterinary uses against a particular disease condition, and *Iu* is the total number of key informants who independently suggested that the same plant or nonplant material has any ethnoveterinary uses.

## 3. Results

The traditional botanical and nonbotanical ethnoveterinary practices used in managing CBPP and other cattle disease conditions as well as the modes of their preparation and administration are presented in Table 1. The traditional botanical and nonbotanical ethnoveterinary practices used in managing other cattle disease conditions as well as the modes of their preparation and administration are presented in Table 2. The local names of plants and nonplant materials in* Hausa*,* Fulfulde*, and* Nupe* were obtained for easy identification and documentation (Table 3).

Traditional preventive practices in use specifically for prophylaxis against CBPP and some cattle disease conditions are shown in Table 4.

## 4. Discussion

Cattle-rearing is the main occupation of Fulani pastoralists in Nigeria and these herdsmen use medicinal plant remedies to manage their stocks [22]. This study indicates that 50 medicinal materials and seven preventive practices are in use by Fulani pastoralists to traditionally manage CBPP and other cattle disease conditions in Niger State. This agrees with earlier reports on the relevance of different traditional healing practices in Nigeria as well as other parts of the world [9, 10, 22]. The reliance of pastoralists on herbal and nonherbal materials for both therapeutic and prophylactic purposes in Nigeria has been reported [13, 22]. The Fulani pastoralists exhibited good existing veterinary knowledge of the pathology of various probed cattle diseases and conditions and the corresponding ethnoveterinary remedies which are mostly acquired from their parents and during grazing. This is in consonance with an observation that the understanding of animal diseases by pastoralists is partly due to experiences gathered during grazing [23].

*A. digitata* (baobab) is commonly found in the northern part of Nigeria and Fulani group frequently uses it in treating CBPP and diarrhea cases in cattle. The study found commonly used medicinal plants by the Fulani pastoralists in the treatment of CBPP cases to include* Adansonia digitata*,* Anogeissus leiocarpus*,* Stachytarpheta angustifolia*,* Striga hermonthica*, and* Terminalia macroptera*. However, it was observed that ethnobotanical management of CBPP is not very effective as indicated by their fidelity levels of the mentioned plants. Except for* A. digitata* and* Terminalia macroptera* that have high fidelity levels, others have very low fidelity levels which may indicate low efficacy of the plants against the disease. The survey revealed that the preventive measures involve the use of lung tissues from infected dead cattle with CBPP (believed to be rich in infective agents) soaked in fresh milk and briefly placed on the nasal area of the healthy ones or wrapped in a rag and hung on a tree very close to the herd site. Also revealed is the application of ground dried infected lungs, by spread of granules in the herd. This traditional immunization finding agrees with earlier reports that livestock keepers are aware of the fact that the principle of vaccination consists of introducing a mild form of the disease [24]. Long ago, many pastoral societies of Africa, such as Maasai, Mauritanian Moors, Somali, and Wodaabe, invented their own vaccines for contagious bovine pleuropneumonia, rinderpest, foot-and-mouth disease, and bovine brucellosis. They used lung tissues, urine, faces, milk, material from the feet, and tongue of the infected animals and material from the aborted fetus to vaccinate other healthy animals [25]. The mention of other preventive practices by the pastoralists agrees with reports that, in other ethnoveterinary medical practices with surgical implications, wounds, joint conditions, and swellings are treated by applying a red-hot iron over them, with the belief that as the burnt skin heals, the ailment is healed along with it [26].

Some of the nonplant materials observed in this survey to be used by the pastoralists include wood ash, honey, oils, kerosene, kaolin, potassium, local soap, and spent engine oil which they believe are effective in ethnoveterinary management. They use spent engine oil in the management of wounds, kerosene for foot rot, and local soap as disinfectant in animals. Some authors [14] have contrast views with the findings as they reported most of these nonplant materials to be carrier mechanisms with no known medicinal values but can cause perceived improvement in performance through their effects on feed efficiency. Further, these authors also observed that the use of a carrier mechanism in ethnoveterinary medical practices involves arbitrary quantities of the carrier which may dilute the drug or reduce its relative potency unlike in conventional veterinary medicine where variability in the quantity of the carrier materials is not much prominent as in ethnoveterinary medicine.

The study found honey to be used in wound healing, oils (especially vegetable oil) for managing poisons and bloats, cow butter for wound healing, cattle fats for burns, and salts for preservation and appetite promotion. These observations have been corroborated by Abdu et al. [22], while Poonam and Singh [27] reported some of them, such as honey, cow/goat's milk, sugar, ghee, salt, and butter milk, to be appetizers media to improve palatability and medicinal property of certain herbal remedies.

The Fulani pastoralists' methods for ethnoveterinary preparation vary and include grinding or pounding dried or fresh parts, followed by boiling or soaking in water to obtain solutions that are administered orally and sometimes mixed with feed. However, ground plant portions could also be mixed with potash or salt and given for licking. These practices of medicinal herbal preparations and administration have been agreed upon by observations of some researchers [14, 22, 28].

The dosage administered often varied with the parts of the plant used and the mode of preparation. However most Fulani pastoralists administer the preparations once or twice daily for a week or keep treating until the animal recovers. Full recovery is confirmed when the animals resume feeding and other physical activities. In a similar observation, Alawa et al. [14] indicated that the duration of treatment for a particular disease in ethnoveterinary practices varied and depends largely on the herdsmen, with clinical improvement of affected animals usually considered as end of that disease condition when they start feeding, leaving the possibility that those causative agents might not be completely eliminated at the beginning of improvement. This contrasts the conventional veterinary medical practices where treatment might continue to complete the dosage even after the clinical signs of a disease have disappeared.

Also, these findings indicate that ethnoveterinary practices are readily available and can complement conventional veterinary medical practices, but there is need to standardize modes of preparation and application of the traditional practices. Further research on the active ingredients and their quantities in the ethnoveterinary materials becomes scientifically necessary so as to guide their usage.

## 5. Conclusion and Recommendations

The information obtained from Fulani pastoralists on ethnoveterinary practices in this study will form a basis for further ethnoveterinary research especially in studies dealing with efficacy, dosage, quality, and toxicology. Those plants that are found to be effective empirically can be used in the preparation of commercial local-based veterinary pharmaceuticals, which will consequently lead to protection of the important ethnoveterinary phytotherapeutics. Since some of the plants used in ethnoveterinary management of cattle by this group of pastoralists are likely to be threatened species, especially with desert encroachment into the state, conservation of such plants is recommended. The Fulani pastoral communities in Niger State are potential beneficiaries of such conservation effort and should be involved in such efforts in the spirit and goal of participatory epidemiology.

## Figures and Tables

**Table 1:** Medicinal plants used by Fulani pastoralists for traditional management of CBPP in Niger State, Nigeria.

S/number	Botanical/scientific name (family)	Common (English) name	Parts used and methods of preparation and application	Disease condition/clinical signs	Fidelity level (100%)
1	*Adansonia digitata* L. (Bombacaceae)	Baobab monkey-bread tree	Grind dried leaves, boil and add potash, and then drench	CBPP (as well as diarrhea)	84
2	*Anogeissus leiocarpus * (DC.) Guill. & Perr. (Combretaceae)	Axlewood	Soak crushed dried leaves with their stems in water and drench	CBPP and diarrhea	26.7
3	*Striga hermonthica * (Del.) Benth. (Scrophulariaceae)	Witch Weed	Soak fresh crushed barks in water and drench	CBPP	23.1
4	*Stachytarpheta angustifolia *Vahl. (Verbenaceae)	Devil's coach whip	Boil whole fresh plant, mix with potash, and drench	CBPP	20.0

**Table 2:** Medicinal plants used by Fulani pastoralists for traditional management of other cattle disease conditions in Niger State, Nigeria.

	Botanical/scientific name (family)	Common (English) name	Local name	Parts used and methods of preparations and applications	Disease condition
1	*Acacia nilotica *(L.) Willd ex Del. (Fabaceae)	Gum arabic	*Bagaruwa* ^*H*^ *Gabdi* ^*F*^, *Gabaruwa* ^*N*^	Pound fresh bark, soak in water, and add red potash. Drench or wash affected areas	Trypanosomosis and foot rot
2	*Allium sativum* L. (Liliaceae)	Garlic	*Tafarnuwa* ^*H*^ *Albasa* *badejo* ^*F*^ *Tafarnuwa* ^*N*^	Mix crushed rhizomes with maize bran and feed as ration	Fascioliasis
3	*Annona senegalensis* Pers. (Annonaceae)	Soursop	*Gwandar* *daji* ^*H*^ *Dukkuhi* ^*F*^ *Nungberechi* ^*N*^	Boil crushed fresh leaves. Wash the wound with warm decoction	Wound
4	*Arachis hypogea * L. (Papilionaceae)	Groundnut	*Gyada* ^*H*^, *Biriji* ^*F*^, *Guzhia* ^*N*^	Oil extract from seeds. Drench	Poisoning
5	*Azadirachta indica *A. Juss. (Meliaceae)	Neem tree	*Dogonyaro* ^*H*^ *Debchi* ^*F*^, *Nimu* ^*N*^	Boil fresh leaves, drench, bath, or wash appropriately	Gastrohelminthiasis, flies infestation, and wound
6	*Bombax buonopozense *P. Beauv. (Bombacaceae)	Red silk, cotton tree	*Gurjiya* ^*H*^ *Bududi* ^*F*^ *Kutukpachi* ^*N*^	Soak ground dried barks in warm water and drench	Trypanosomosis
7	*Butyrospermum paradoxum* (Gaertn.f.) Hepper (Sapotaceae)	Shea butter tree	*Kadanya* ^*H*^ *Kochi* ^*N*^ *Karaji* ^*F*^	Crush seeds, extract oil, and apply topically	Dermatophilosis
8	*Cassia occidentalis *Linn. (Caesalpiniaceae)	Coffee senna	*Tafasar* *masar* ^*H*^ *Tapasa* ^*F*^, *Gaya* ^*N*^	Boil fresh leaves, add salt, and drench the concoction	Gastrohelminthiasis
9	*Citrus aurantifolia *(Christm.) Swingle (Rutaceae)	Sour orange, sour lime	*Lemun* *tsami* ^*H*^ *Lammude* ^*F*^ *Lemu* *bakagi* ^*N*^	Add red potash and the juice to water. Mix and drench with the concoction	Brucellosis
10	*Crossopteryx febrifuga *(Afzel. ex G. Don) Benth. (Rubiaceae)		*Kasfiya* ^*H*^ *Nambi* *susun* ^*N*^	Boil crushed fresh leaves and barks. Add potash and drench	Brucellosis
11	*Dichrostachys glomerata* (Forsk.) Chiov. (Fabaceae)	Cow thorn	*Dundu* ^*H*^, *Burli* ^*F*^ *Ekan*-*nanko* ^*N*^	Boil fresh leaves and wash the wound with warm solution	Wound
12	*Dissotis rotundifolia *(Sm.) Triana (Melastomataceae)		*Edingi*-*bata* ^*N*^	Boil fresh leaves and drench	Trypanosomosis
13	*Entada africana *Guill. & Perr. (Fabaceae)		*Tawatsa* ^*H*^ *Peluwahi* ^*F*^ *Kawo*-*nuwanchi* ^*N*^	Boil crushed barks. Drench and also apply topically. Boil crushed barks. Drench and also apply topically	FMD
14	*Khaya senegalensis *(Desr.) A. Juss. (Meliaceae)	Mahogany	*Khaya senegalensis* (Desr.) A. Juss. (Meliaceae)	Boil dried barks, add potash, and drench. Crush fresh bark to paste and apply topically	Brucellosis, dermatophilosis, diarrhea, bloat, foot rot, and poisoning
15	*Kigelia africana *(Lam.) Benth. (Bignoniaceae)	Sausage	*Rawuya* ^*H*^ *Jillarehi* ^*F*^, *Bechi* ^*N*^	Boil crushed dried barks and cool. Add salt and drench	Brucellosis
16	*Lawsonia inermis *(L.) Keay (Lythraceae)	Henna plant	*Lalle* ^*H*^ *Poldi* ^*F*^, *Lali* ^*N*^	Boil fresh leaves and drench	Fasciolosis
17	*Mitracarpus scaber *Zucc. and ex Schult + Schult.f. (Rubiaceae)		*Harwatsi* ^*H*^, *Yikunu*-*kparagi* ^*N*^	Grind dried leaves. Mix powder with cow butter oil and apply topically	Dermatophilosis
18	*Ocimum gratissimum *L. (Lamiaceae)	Basil fever plant	*Daidoya* *ta* *gida* ^*H*^ *Tanmotswagi* ^*N*^	Pound fresh leaves into paste and apply on affected areas topically	Wound
19	*Parkia biglobosa * Aubrevielle (Fabaceae)	African locust bean	*Dorowa* ^*H*^ *Narehe* ^*F*^, *Lonchi* ^*N*^	Grind dried roots, soak, and administer decoction orally	Diarrhea
20	*Piliostigma thonningii * (Schum.) Milne-Redhead (Caesalpiniaceae)	Thonning's pilostigma	*Kalgo* ^*H*^ *Bartehi* ^*F*^, *Bafin* ^*N*^	Grind fresh or dried bark, mix with any type of bran, and feed as ration	Diarrhea
21	*Prosopis africana *(Guill. & Perr.) Taub. (Fabaceae)	Guava	*Kirya* ^*H*^, *Kohi* ^*F*^ *Sanchi* ^*N*^	Boil fresh leaves and stems, add potash, and drench	Diarrhea
22	*Psidium guajava *L. (Myrtaceae)		*Goiba* ^*H*^ *Goyiba* ^*N*^ *Kwashi* ^*F*^	Soak ground leaves in water. Add red potash and drench	Diarrhea
23	*Lophira lanceolata* Van Tiegh. ex Keay (Ochnaceae)	Iron wood	*Namijin*-*kade* ^*H*^ *Maganci* ^*N*^ *Kochi*-*kere* ^*N*^ *Karereshi*-*yolde* ^*F*^	Crush dried barks and mill into powder with shea butter. Apply topically	Dermatophilosis and ticks infestation
24	*Ricinus communis *Linn. (Euphorbiaceae)	Castor oil tree	*Dan* *kwasare* ^*H*^ *Kolakolahi* ^*F*^ *Kpanfiniko*-*gulu* ^*N*^	Crush fresh leaves to paste. Apply paste on affected areas topically	Dermatophilosis and wounds
25	*Sarcocephalus latifolius *(Sm.)Bruce (Rubiaceae)	African peach	*Tafashiya* ^*H*^ *Gbashi* ^*N*^ *Bakureshi* ^*F*^	Boil fresh roots. Add one tea spoonful salt and drench	Mastitis
26	*Schwenckia americana *Linn. (Solanaceae)		*Dandan* ^*H*^ *Dandana* ^*F*^ *Kabi*-*malam* ^*N*^	Boil crushed dried barks and add potash. Drench	Bloat and mastitis
27	*Senna (Cassia) alata *(L.) Roxb. (Caesalpiniaceae)	Ringworm plant, craw- craw plant	*Gungoroko* ^*N*^	Grind dried leaves into powder. Mix with cow butter oil and apply topically	Dermatophilosis, wound, and ringworm
28	*Tephrosia vogelii *Hook.f. (Fabaceae)	Fish-poison bean, Vogel's tephrosia	*Magamun* ^*H*^ *Yomji* ^*F*^ *Egga* ^*N*^	Grind dried leaves and stems. Dissolve powder in water and bath the affected cattle	Lousiness and ticks infestation
29	*Terminalia macroptera* Guill. & Perr. (Combretaceae)		*Baushe* ^*H*^ *Bodihi* ^*F*^, *Kpace* ^*N*^	Crush dried roots and boil. Add potash and drench	Diarrhea
30	*Vernonia amygdalina *Del. (Asteraceae)	*Vernonia amygdalina *Del. (Asteraceae)	*Shiwaka* ^*H*^ *Shuwaka* ^*F*^ *Tsula* ^*N*^	Soak crushed fresh leaves. Add potash and drench	Gastrohelminthiasis
31	*Vitex doniana *Sweet (Verbenaceae)	Black plum	*Dinya* ^*H*^ *Bodilohi* ^*F*^ *Dinchi* ^*N*^	Crush fresh barks, soak in water, and drench	FMD, diarrhea, and retained placenta
32	*Zanthoxylum zanthoxyloides *Zepernick & Timler. (Rutaceae)	African satinwood	*Fasakuwari* ^*H*^ *Fasakwabri* ^*F*^ *Kosonkori* ^*N*^	Boil dried barks and drench	Trypanosomosis
33	*Zingiber officinale* Rosc. (Zingiberaceae)	Ginger	*Tsita* *mai* *yatsu* ^*H*^, *Tsutafu* ^*N*^	Crush dried rhizomes, mix with maize bran, and feed as ration	Diarrhea
34	*Ziziphus abyssinica *Hochst. ex A. Rich. (Rhamnaceae)	Catch thorn	*Magariya* *kura* ^*H*^ *Jabe* *puri* ^*F*^ *Dangodi* ^*N*^	Boil crushed fresh leaves and drench or wash wound surface	Diarrhea and wound

Superscript letters *H*, *F*, and *N* represent *Hausa*, *Fulani,* and *Nupe * languages, respectively.

**Table 3:** 

S/number	Materials	Local name	Ethnoveterinary uses	Fidelity levels (%)
1	Limestone		Decoction and concoction	92.3
2	Honey	*Zuma* ^*H*^	Wound healing and preservative	100.0
3	Oil	*Mai* ^*H*^	Vegetable oil is used in managing poisons and bloats. It can also be used as preservative	100.0
4	Cow butter	*Mai*-*shanu* ^*H*^	Wound healing and preservative	96.3
5	Salts		Preservative and appetite promotion	100.00
6	Used (spent) engine oil	*Bakin mai* ^*H*^	Treatment of many skin conditions (e.g., wound, dermatophilosis, mange, and ringworm)	78.3
7	Local potassium (potash)	*Kanwa* ^*H*^	Part of decoction to relieve bloat, diarrhea, mastitis; mix with used engine oil to treat dermatophilosis	100.0
8	Cattle fats		Preservatives and treatment of burns	83.3
9	Wood ash		Preservative and disinfectant, specifically for managing foot rot, and its paste rub on cow genital area to induce expulsion of placenta	100.0
10	Kerosene	*Kanazine* ^*H*^	Used to wash foot rot area to hasten its healing	88.5
11	Kaolin		Treating diarrhea	100.0
12	Local soap		Treatment of ringworm Treatment of ringworm	86.7

Note: superscript letters *H*, *F*, and* N* represent local names in *Hausa, Fulfulde,* and *Nupe,* respectively.

**Table 4:** Traditional prophylactic (preventive) practices used by Fulani pastoralists for the management of CBPP and other cattle disease conditions in Niger State, Nigeria.

S/number	Preventive practices	Modes of preparations and applications	Ethnoveterinary uses	Fidelity level (%)
1	Vaccination	Lung tissues from infected dead cattle with CBPP (believed to be rich in infective agents) are soaked in fresh milk and briefly placed on the nasal area of the healthy ones or wrapped in a rag and hung on a tree very close to the herd site. Also dry the lung and grind and spread the granules in the herd	Preventive measure against CBPP	56.5

2	Vaccination	Tissue materials from the feet and tongue of the infected cattle or saliva used on healthy ones or feces of infected cattle on the feet and mouth areas of healthy ones	Preventive measure against foot and mouth disease (FMD)	33.3

3	Vaccination	Fluid of aborted fetuses, mixed with urine and rubbed on the genital and udder areas	Prophylactic measure against bovine brucellosis	46.2

4	Repellant	Burning of dried grasses or dried wood at the mid of herds in the morning before going on grazing and immediately on return from grazing in the evening	To repel biting and sucking flies	100.0

5	Branding	Sharp iron is inserted into fire until it reddens. It is then removed and two straight lines are engraved parallel to or across each other on the swollen area	To relieve inflammation due to trauma and treat black quarter, lameness, rheumatic complex, and some skin diseases	75.0

6	Grooming	Use of fingers or hard brush to groom the skin periodically	To remove fleas and lice	88.2

7	Herd size sanitation	Frequent removal of feces from herd site base and also grazing areas	To reduce greatly the parasitic burden	100.0
